# Gluteal muscle activation during rehabilitation exercises in female field hockey players

**DOI:** 10.4102/sajp.v77i1.1578

**Published:** 2021-10-01

**Authors:** Daretha Coetzee, Frederik F. Coetzee, Robert Schall, Colleen Sinclair

**Affiliations:** 1 Department of Exercise and Sport Sciences, Faculty of Health Sciences, University of the Free State, Bloemfontein, South Africa; 2 Department of Mathematical Statistics and Actuarial Science, Faculty of Natural and Agricultural Sciences, University of the Free State, Bloemfontein, South Africa

**Keywords:** field hockey, high-performance, female, body weight rehabilitation exercises, maximal voluntary isometric contraction, MVIC, gluteus maximus, gluteus medius, surface electromyography

## Abstract

**Background:**

Field hockey, a team sport played by both men and women at both recreational and professional levels, requires maintaining a forward flexed posture putting stress on the lumbar spine. Hence, it is necessary to assess the muscles supporting the lumbar spine, especially those surrounding the hip, to inform strengthening exercises for this population.

**Objectives:**

To establish the best body weight rehabilitation exercises shown to produce high muscle activation (≥ 61%MVIC – maximal voluntary isometric contraction) for both the gluteus maximus (Gmax) and medius (Gmed) muscles. Four exercises fell into this category.

**Method:**

Surface electromyography (sEMG) was used to record the muscle activation of Gmax and Gmed of four body weight rehabilitation exercises in 26 high-performance female field hockey players. The %MVIC activation data of both Gmax and Gmed were analysed using a three-way ANOVA.

**Results:**

The single-leg squat generated the highest %MVIC activation of both Gmax (125.65%MVIC) and Gmed (126.30%MVIC). The only statistically significant difference for Gmax was between the single-leg squat and plank with hip extension (*p* = 0.0487). No statistically significant difference was observed for Gmed between the four body weight rehabilitation exercises (*p* = 0.6285).

**Conclusion:**

The four exercises generated similar %MVIC activation levels. The single-leg squat produced the highest observed %MVIC of Gmax and Gmed in high-performance female field hockey players and is, therefore, recommended.

**Clinical implications:**

Implementation of the findings could result in benefits during prehabilitation, injury prevention programmes and the later stages of rehabilitation for high-performance female field hockey players.

## Introduction

Field hockey is a non-gender specific international team sport played at many levels, ranging from amateur to elite (Jennings et al. 2012; McGuinness et al. 2019). The synthetic surfaces on which high-performance field hockey is played predominantly demand maintaining a forward flexed posture (semi-crouched/squatting) to optimally execute skills such as stopping, flicking and tackling (Haydt, Pheasant & Lawrence 2012; Wege et al. 2006). Hence, the postural stresses on the musculoskeletal system during play on these surfaces can cause the development of lower back pain in this population (Haydt et al. 2012; Wege et al. 2006). Wege et al. (2006) states that the muscles inferior to the pelvis (the hip and knee joint musculature) and the muscles superior to the pelvis (the lumbar spine and abdominal wall musculature) control the forces acting on the lumbar spine. Considering the postural requirements of field hockey and its influence on a player’s spine, the muscles that support the lumbar spine, especially the muscles surrounding the hip, need to be assessed (Wege et al. 2006).

The hip muscles, in particular gluteus maximus (Gmax), play a significant role in transferring forces from the lower extremity in the direction of the spine. It decreases the load on the erector spinae during lumbar extension and stabilises the knee in extension (Neumann 2010; Stegeman & Hermens 2014). However, Gmax is prone to weakness, which results in reduced stability of the lumbar spine and sacroiliac joint (Wege et al. 2006). Wege et al. (2006) and Bishop et al. (2018) report an association between female field hockey players with weak Gmax and chronic lower back pain. A weak gluteus medius (Gmed) could disrupt movement and may lead to adverse alterations in lower extremity kinematics that increase the injury risk in players and result in deterioration in sports performance (Stastny et al. 2016). Gmed acts primarily by producing abduction at the hip joint and is critical for pelvic and lower limb (femur) stability during weight-bearing movements, such as the different actions required in field hockey (Ebert et al. 2017; Reiman, Bolgla & Loudon 2012; Stegeman & Hermens 2014; Van Putte et al. 2014).

Weakness of Gmax and Gmed will result in compensatory movements of the lower back, hip and knee, notably a pelvic drop, excessive hip adduction, femoral internal rotation and an exaggerated knee valgus angle (Distefano et al. 2009; Macadam, Cronin & Contreras 2015; Reiman et al. 2012). Against this background, the hip extensors and abductors that support the lumbar spine structures of high-performance female field hockey players warrant investigation.

In recent years, various studies have indicated that structured exercise is the first step towards injury prevention in team sports (Barboza et al. 2018). However, a limited number of studies on the implementation of this kind of programme in field hockey have been reported. Therefore, exercise programmes that have been shown to be effective in preventing lower limb injuries should be introduced regularly to field hockey teams as part of their training schedule (Barboza et al. 2018). Furthermore, although various gluteal strengthening exercises are available, a thorough knowledge of which exercises optimally target Gmax and Gmed, as well as the magnitude of activation related to each exercise, needs to be established (Macadam et al. 2015). Studies by Escamilla et al. (2010) and Ebert et al. (2017) concerning the classification of low and high muscle activity, categorised between 0% and 20% maximal voluntary isometric contraction (MVIC) as ‘low’ muscle activity, 21% to 40%MVIC as ‘moderate’, 41% to 60%MVIC as ‘high’ and more than 61%MVIC as ‘very high’ muscle activity.

Our study investigated the activation capabilities of Gmax and Gmed during various rehabilitation exercises documented in previous studies, to establish which commonly prescribed body weight rehabilitation exercise produced greater than 61%MVIC for both Gmax and Gmed. Only body weight rehabilitation exercises that are performed without any additional external load, such as a barbell, dumbbell, band and machine, as well as plyometric or hopping movements, were considered. The only studies that met these criteria were those of Boren et al. (2011), Simenz et al. (2012), Webster and Gribble (2013), Lee et al. (2014), MacAskill, Durant and Wallace (2014), Kim and Yoo (2015), Jeon, Kwon and Weon (2016), Ebert et al. (2017), Macadam and Feser (2019) and Cochrane, Gabriel and Harnett (2019). Cochrane et al. (2019) was the only study conducted in a high-performance population, namely rugby players. The remaining studies selected apparently healthy individuals. Boren et al. (2011) published the only study that reported sufficient %MVIC for both Gmax and Gmed during four exercises. These exercises were side-plank hip abduction with the dominant leg at the bottom (70.96%MVIC of Gmax; 103.11%MVIC of Gmed), side-plank hip abduction with the dominant leg on top (72.87%MVIC of Gmax; 88.82%MVIC of Gmed), the single-leg squat (82.26%MVIC of Gmax; 70.74%MVIC of Gmed) and plank with hip extension (106.22%MVIC of Gmax; 75.13%MVIC of Gmed). Our examined these four exercises on high-performance female field hockey players of a University in South Africa.

The aim of our study was to examine the activation capabilities of the gluteal muscles during four rehabilitation exercises and, in particular, to determine which exercise elicited the highest %MVIC for both Gmax and Gmed in high-performance female field hockey players.

## Methods

A randomised, 4-period, 4-treatment crossover trial of four body weight rehabilitation exercises, compared the activation capabilities of the gluteal muscles in high-performance female field hockey players.

We included apparently healthy females who were part of the University of the Free State (UFS) high-performance field hockey squad and consented to participate. Players with self-reported lower back, lower extremity or pelvic girdle injuries or a history of lower extremity surgery within the 2 years preceding our study were excluded. Two cohorts of 26 participants in total were recruited for the trial. The first cohort of 14 participants was tested during October 2019, while the second cohort of 12 participants was tested in February 2020.

### Selected exercises

The four body weight rehabilitation exercises conducted by Boren et al. (2011) that were tested included (1) side-plank hip abduction with the dominant leg at the bottom; (2) side-plank hip abduction with the dominant leg on top; (3) the single-leg squat and (4) plank with hip extension. These exercises produced the highest %MVIC in a healthy population according to published reports (Boren et al. 2011; Ebert et al. 2017; Escamilla et al. 2010; Han et al. 2018; Jeon et al. 2016; Kendall et al. 2005; Macadam & Feser 2019; McBeth et al. 2012; Tabard-Fougère et al. 2018).

#### Side-plank hip abduction with dominant leg at the bottom

The participant started in a side-lying position with the dominant leg on the bottom. The lowermost leg was flexed to 45° at the hip and knee to assist in stabilising the pelvis against anterior or posterior tilting (Kendall et al. 2005; Tabard-Fougère et al. 2018). The participant subsequently rose to a plank position with the hips raised from the plinth, whilst being allowed to use the upper extremity for support (Boren et al. 2011). The first author instructed the participant to abduct the non-dominant leg to measure a 35° hip abduction angle and adjusted a hurdle target bar accordingly to ensure that the participant executed the exercise within the 35° angle (Ebert et al. 2017; McBeth et al. 2012). Finally, the participant was instructed to abduct the non-dominant leg for two beats and then lower the leg for two beats, whilst maintaining the side-plank position throughout all repetitions (Boren et al. 2011). The participant’s foot touched the hurdle bar on the second beat upwards in abduction and the plinth on the second beat downwards in adduction to complete one repetition.

#### Side-plank hip abduction with dominant leg on top

The participant started in a side-lying position with the dominant leg on top and was instructed to flex the lowermost leg at the hip and knee to 45° (Kendall et al. 2005; Tabard-Fougère et al. 2018). Thereafter, she was asked to rise to a plank position with the hips raised from the plinth, whilst being allowed to use the upper extremity for support (Boren et al. 2011). The first author instructed the participant to abduct the dominant leg to measure a 35° hip abduction angle and adjusted a hurdle target bar to ensure that the participant executed the exercise within the 35° angle (Ebert et al. 2017; McBeth et al. 2012). Finally, the participant was instructed to abduct the dominant leg for two beats and then lower the leg for two beats, whilst maintaining the side-plank position throughout all repetitions (Boren et al. 2011). The participant’s foot touched the hurdle bar on the second beat upwards in abduction and the plinth on the second beat downwards in adduction to complete one repetition.

#### The single-leg squat

The participant was requested to stand on the dominant leg, 15 cm away from a chair (used for standardisation amongst all participants), with the knee of the non-dominant leg extended and the hip flexed to avoid the heel of the contralateral leg touching the floor during the execution of the exercise. The participant was then asked to flex the dominant leg’s knee for two beats, touch a chair of 47 cm in height with the buttocks and extend back to the upright position for two beats (Boren et al. 2011). A mirror was placed in front of the participant to provide visual feedback of the (correct) position of the head and trunk, and to prevent tilting of the trunk towards the weight-bearing leg to compensate for Gmed weakness throughout the execution of the exercise (Han et al. 2018).

#### Plank with hip extension

The player started prone on their elbows in a plank position with the trunk, hips and knees in neutral alignment (Boren et al. 2011). The participant was requested to lift the dominant leg off the plinth to perform a prone active straight leg raise from the plank position and then flex the knee of the dominant leg. This was followed by elevating the heel towards the ceiling in a kick back action to extend the hip. The first author measured a hip extension angle of 5° and a knee flexion angle of 90° and adjusted the hurdle target bar accordingly (Jeon et al. 2016). At the start of the exercise, she instructed the participant to perform a dominant leg lift in a position of 90° knee flexion (Macadam & Feser 2019). Thereafter, the participant initiated the movement action from 30° hip flexion towards 5° hip extension for two beats and then slowly returned the dominant leg to 30° hip flexion for two beats to complete one repetition (Jeon et al. 2016; Macadam & Feser 2019). To ensure that the participant maintained the correct hip flexion angle, the hip flexion angle was measured in such a way that the participant should touch the plinth with her dominant leg’s knee at the end of the repetition.

Each player commenced with the four body weight rehabilitation exercises after the execution of the MVIC trials. The participants had a 2-min rest period between the different exercises. The exercise order was randomised to avoid order bias, for example, because of fatigue (Boren et al. 2011). The first author measured specific range of motion angles with a universal goniometer for standardisation among participants, and to eliminate activation of adjacent muscles to primarily activate Gmax and Gmed (Jeon et al. 2016). Furthermore, consistent verbal feedback was provided to ensure the optimal execution of each exercise (Boren et al. 2011).

To determine the %MVIC produced by the exercises, each participant executed eight repetitions of each exercise. The first three repetitions were for practice and familiarisation purposes, whereas the last five repetitions were recorded (Boren et al. 2011). The highest peak surface electromyography (sEMG) signal out of these repetitions was then divided by the normalised MVIC value to calculate the %MVIC. The peak %MVIC of each exercise was averaged amongst all the participants to report the findings of each muscle.

### Testing procedure

The participants reported to the UFS Exercise and Sport Sciences Centre for the testing procedure. The first author tested each player, who met the inclusion criteria with the assistance of a registered biokineticist, who recorded the sEMG activity. The first author explained the testing procedures and the content of documents to the participants. The health screening form obtained the following information: age, dominant leg, exercise frequency, type of physical activity, injury history and a self-reported lower quarter screening to identify exclusion criteria. The first author then measured and documented the participant’s weight and height according to the anthropometric guidelines of the World Health Organization (WHO 2017).

The participant warmed up for 5 min on a Wattbike cycle ergometer (Wattbike; Nottingham, United Kingdom) whilst the first author demonstrated the MVIC testing positions and the four exercises. She prepared and cleaned the skin. Kendall Meditrace Ag/AgCl pre-gelled electrodes (Cardinal Health; Mansfield, United States of America) were used to reduce electrode-skin impedance (Konrad 2006; Merletti 2015; Stegeman & Hermens 2014). The intention of skin preparation was to achieve constant electrode contact and low skin impedance through appropriate fixation of the electrodes (Konrad 2006). Concordia University PERFORM Centre (2016) and Stegeman and Hermens (2014) confirmed that alcohol and a gauze pad were sufficient for cleaning the surface of the skin. As described by Stegeman and Hermens (2014), the alcohol was allowed to vaporise for the skin to be dry before the placement of the electrodes. The first author marked the electrode sites on the participant’s skin and placed the electrodes following the sEMG for non-invasive assessment of muscles (SENIAM) guidelines (Stegeman & Hermens 2014), as summarised in Table 1.

**TABLE 1:** Surface EMG for non-invasive muscle assessment guidelines for surface electromyography.

Variable	Gluteus maximus (Gmax)	Gluteus medius (Gmed)
Starting posture	Lying down in a prone position on a plinth	Side-lying on a plinth.
Electrode size	10 mm in the direction of the muscle fibres orientation	10 mm in the direction of the muscle fibres orientation.
Electrode distance	20 mm	20 mm
Location	Half-way between the sacrum and greater trochanter – greatest prominence of the middle of the buttocks	Half-way between the iliac crest and greater trochanter
Orientation	Directly in line with the PSIS and the posterior aspect of the thigh	Directly in line with the iliac crest and greater trochanter
Fixation on the skin	Use double-sided tape or elastic bands	Use double-sided tape or elastic bands

*Source*: Stegeman, D.F. & Hermens, H.J., 2014, *Standards for surface electromyography: The European project `Surface EMG for non-invasive assessment of muscles (SENIAM)*’, viewed 24 February 2021, from https://www.researchgate.net/profile/Hermie_Hermens/publication/228486725_Standards_for_suface_electromyography_The_European_project_Surface_EMG_for_non-invasive_assessment_of_muscles_SENIAM/links/09e41508ec1dbd8a6d000000/Standards-for-suface-electromyography-The-European-project-Surface-EMG-for-non-invasive-assessment-of-muscles-SENIAM.pdf SENIAM, surface electromyography for non-invasive muscle assessment; PSIS, posterior superior iliac spine.

### Maximal voluntary isometric contraction normalisation

Participants adopted the MVIC testing positions for normalisation of the sEMG signal amplitude consisting of a 5 s isometric hold, 3 × repetitions, 2 min’ rest in between. Normalisation of the sEMG signal amplitude is important because of various influences on the sEMG signal, such as individual differences and anatomical and physiological factors (Cho, Kim & Park 2018). A standardised assessment method is required through the execution of a manual muscle strength test as a reference contraction to determine the MVIC of a particular muscle before executing each exercise (Cho et al. 2018; Huseth et al. 2020; Lee & Jo 2016). The highest peak value from the three trials recorded of the manual muscle strength tests for Gmax and Gmed was used to determine the MVIC.

Kendall et al. (2005), McBeth et al. (2012), Webster and Gribble (2013), Kim and Yoo (2015) and Jeon et al. (2016) recommended that the manual muscle strength test for the Gmax should be executed in hip extension with 90° knee flexion, thus confirming it as the general position used to establish MVIC of Gmax. Jeon et al. (2016) and Macadam and Feser (2019) showed that hip extension should be 5° to achieve optimal activation of Gmax. Therefore, we executed the manual muscle strength test in a position of 90° knee flexion and 5° hip extension through an isometric contraction of 5 s, repeated three times, with 2 min of rest in between (Boren et al. 2011; Halaki & Ginn 2012; Jeon et al. 2016).

McBeth et al. (2012) recommended that the manual muscle strength test for Gmed should be executed in a position of hip abduction to approximately 35°, slight hip extension and medial rotation of the hip. Ebert et al. (2017) reported that a medially rotated hip position elicited higher Gmed activity compared to a laterally rotated hip position that produced significant tensor fascia latae (TFL) activity. Three repetitions of a 5-s isometric contraction separated by 2 min of rest between each isometric contraction were used to determine the MVIC (Boren et al. 2011; Halaki & Ginn 2012; Jeon et al. 2016).

### Data processing

The sEMG signal amplitude was normalised to MVIC and was measured for both the Gmax and Gmed. All data were rectified using a root mean square (RMS) algorithm and smoothed with a time reference. The sEMG bandwidth was 20 Hz – 450 Hz, common-mode rejection ratio (CMRR) >100 decibels (dB) at 60 Hz, input impedance > 100 electrical resistance and conductance (Ohm), baseline noise < 1 potential difference (µV) RMS, a sufficient sEMG signal gain of 400 Volts (V) at a sample frequency of 1000 Hz.

### Statistical analysis

The %MVIC activation data of both Gmax and Gmed were analysed using a three-way analysis of variance (ANOVA), with ‘participant’, ‘period’ (referring to the randomised order in which exercises were conducted) and ‘exercise’ as categorical variables in the model. Fitting the ‘participant’ effect also controlled for the cohort effect, namely the potential effect of testing the trial participants in two cohorts. Based on this three-way ANOVA model, point estimates for the mean %MVIC for each exercise were reported, as well as point estimates, 95% confidence intervals (CIs) and *p*-values for the pairwise differences in peak %MVIC between the four body weight rehabilitation exercises. For each variable analysed, the overall F-test for the four body weight rehabilitation exercises was determined, and also the partial effect size measure for the ANOVA (SAS Institute Inc. 2017).

### Ethical considerations

Our study was approved by the Health Sciences Research Ethics Committee (HSREC) of the Faculty of Health Sciences, UFS, South Africa (Ethics No. UFS-HSD2019/1349/2910).

A written informed consent form was provided to and signed by each of the participants that outlined the risks emanating from the procedures administered and the equipment used during our study.

## Results

### Demographic characteristics of the study sample

Twenty-six high-performance female field hockey players of the UFS completed the four body weight rehabilitation exercises. The mean age of the players was 20.15 years (standard deviation [SD] ± 1.59 years, range 18–24 years), the mean height was 164 cm (SD ± 0.07 cm, range 151 cm – 177 cm), mean body mass was 64.72 kg (SD ± 10.21 kg, range 51.2 kg – 87.2 kg) and mean body mass index (BMI) was 23.87 kg/m^2^ (SD ± 2.92 kg/m^2^, range 19.39 kg/m^2^ – 31.58 kg/m^2^).

Table 2 presents the descriptive statistics for Gmax and Gmed activation data, and Table 3 presents the results of the ANOVA. Of the four exercises, the single-leg squat had both the highest average Gmax and Gmed activation. However, as shown in Table 3, the overall F-statistic for ‘exercise’ was not statistically significant for either Gmax (*p* = 0.2558) or Gmed (*p* = 0.6285) activation, and the associated effect sizes for ‘exercise’ were negligibly small. Furthermore, none of the pairwise comparisons of the four exercises with regard to Gmax and Gmed showed statistically significant differences, except for the comparison of the single-leg squat with plank hip extension with regard to Gmax (*p* = 0.0487).

**TABLE 2:** Peak %maximal voluntary isometric contraction activation data of female field hockey players (*n* = 26).

Exercise	Muscle %MVIC activation			
	Gmax (*n* = 26)		Gmed (*n* = 26)	
	Mean	± SD	Mean	± SD
**Side-plank hip abduction**				
Dominant leg at the bottom	124.61	7.94	126.07	14.16
Dominant leg on top	124.33	8.63	124.52	11.37
Single-leg squat	125.64	10.13	126.30	12.89
Plank with hip extension	122.73	9.37	125.04	13.14

MVIC, maximal voluntary isometric contraction; Gmax, gluteus maximus; Gmed, gluteus medius; SD, standard deviation.

**TABLE 3:** Three-way analysis of variance (ANOVA) of gluteus maximus (Gmax) and gluteus medius (Gmed).

Dependent variable	Fixed effect	F-statistic (denominator *df* = 72)*	Numerator (*df*)	*p*	Effect size	
					Partial ω^*2*^	*ω=√ω^2^*
Gmax [%MVIC]	Participant	9.00	25	< 0.0001	-	-
	Period	2.33	3	0.0816	-	-
	Exercise	1.38	3	0.2558	0.0108	0.10
Gmed [%MVIC]	Participant	18.9	25	< 0.0001	-	-
	Period	2.66	3	0.0542	-	-
	Exercise	0.58	3	0.6285	0.0122	0

* , F-statistics, *p*-value and effect size statistic from three-way analysis of variance (ANOVA) model with ‘participant’ and ‘exercise’ as fixed effects.

MVIC, maximal voluntary isometric contraction; *df*, degrees of freedom.

Table 3 shows that the participant effect was statistically significant in the analysis of both Gmax and Gmed activation (*p* < 0.0001). Whilst significant differences among participants were expected, although it was not the focus of our study, those differences presented why Gmax and Gmed activations were studied in a crossover trial where the large between-participant variability could be accounted for in the statistical analysis. ‘Period’ was statistically significant for Gmax (*p* = 0.0816) and Gmed (*p* = 0.0542) at the 10% significance level, again justifying the crossover trial, which allows one to control the period differences in the trial.

Table 4 shows detailed statistics of the pairwise difference between the four body weight rehabilitation exercises of Gmax and Gmed, namely the mean difference, 95% CI for the mean difference and *p*-values associated with the effect of ‘exercise’ on Gmax and Gmed.

**TABLE 4:** Pairwise mean differences between exercises for gluteus maximus and gluteus medius %maximal voluntary isometric contraction.

Variable	Pair of exercises^†^	Mean difference^‡^	95% CI for mean difference^‡^	*p* ^‡^
mGmax [%MVIC]	1 vs 2	0.28	-2.61–3.17	0.8475
	1 vs 3	-1.03	-3.94–1.87	0.4807
	1 vs 4	1.88	-1.02–4.77	0.2000
	2 vs 3	-1.31	-4.21–1.58	0.3685
	2 vs 4	1.60	-1.31–4.50	0.2770
	3 vs 4	2.91	0.02–5.80	0.0487
mGmed [%MVIC]	1 vs 2	1.54	-1.57–4.66	0.3272
	1 vs 3	-0.23	-3.36–2.90	0.8837
	1 vs 4	1.03	-2.09–4.14	0.5134
	2 vs 3	-1.77	-4.89–1.34	0.2606
	2 vs 4	-0.52	-3.64–2.61	0.7437
	3 vs 4	1.26	-1.86–4.37	0.4240

MVIC, maximal voluntary isometric contraction; CI, confidence interval.

† , Exercise 1: side-plank hip abduction with dominant leg at the bottom; Exercise 2: side-plank hip abduction with dominant leg on top; Exercise 3: single-leg squat; Exercise 4: plank with hip extension.

‡ , Mean differences, 95% CI for mean differences and *p*-values (for effect of ‘exercise’) from three-way analysis of variance (ANOVA) model with ‘participant’, ‘period’ and ‘exercise’ as fixed effects.

## Discussion

Whilst various body weight rehabilitation exercises are prescribed clinically to strengthen Gmax and Gmed, no evidence is available on the relative efficacy of these exercises in high-performance female field hockey players. The trial reported here selected exercises that generate a ‘very high’ (> 61%MVIC) muscle activation threshold (Ebert et al. 2017; Escamilla et al. 2010). The only exercises that fell into this category were four body weight rehabilitation exercises evaluated by Boren et al. (2011) and, accordingly, these exercises were selected to be examined through sEMG recording.

### Side-plank hip abduction with dominant leg at the bottom and dominant leg on top

Exercises that involve hip abduction are typically prescribed for strengthening Gmed, with the most common version being side-lying hip abduction (Distefano et al. 2009; Ebert et al. 2017; Reiman et al. 2012). However, we examined a side-plank version of hip abduction, because Boren et al. (2011) found that this version generated the highest %MVIC activation of the primary function of Gmed, namely hip abduction.

Considering the side-plank hip abduction with the dominant leg at the bottom, Gmax on average was 124.61%MVIC and the Gmed 126.07%MVIC (Table 2), whilst Boren et al. (2011) reported a 70.96%MVIC of Gmax and 103.11%MVIC of Gmed. Boren et al. (2011) and Ebert et al. (2017) concluded that 103.11%MVIC exhibited by Gmed was the highest percentage activation and therefore the best exercise to prescribe for strengthening Gmed. On the contrary, we found that side-plank hip abduction with the dominant leg at the bottom was the second-best exercise for strengthening Gmed, although the difference from the best exercise (single-leg squat) results was very small and not statistically significant.

Considering side-plank hip abduction with the dominant leg on top, Table 2 shows average values of 124.33%MVIC for Gmax and 124.52%MVIC for Gmed, whilst Boren et al. (2011) reported 72.87%MVIC and 88.82%MVIC for Gmax and Gmed, respectively. They concluded that compared to all the other exercises examined in their study, the 88.82%MVIC for Gmed was the second-best exercise to prescribe for strengthening Gmed (Boren et al. 2011). In contrast, we found that side-plank hip abduction with the dominant leg on top elicited the lowest activation of Gmed compared to the other three exercises.

Side-plank hip abduction with the dominant leg on top requires that Gmax and Gmed have to overcome the gravity barrier during concentric hip abduction (Lee & Jo 2016), which is in contrast with the dominant leg at the bottom version where Gmax and Gmed function in a stabilising state. Despite this contrast, no significant difference was found between the two versions of side-plank hip abduction in high-performance female field hockey players for both Gmax (*p* = 0.8475, *d* = 0.28) and Gmed (*p* = 0.3272, *d* = 1.54), as shown in Table 4. The %MVIC findings for both side-plank hip abduction versions suggested that Gmax and Gmed activation in high-performance female field hockey players were similar to the unilateral leg in an isometric, stabilising state (the dominant leg is at the bottom) and to the contralateral leg in an isotonic state (the dominant leg is on top) to perform concentric hip abduction. Boren et al. (2011) reported that their healthy participants elicited greater activation of only Gmed during the side-plank hip abduction with the dominant leg at the bottom, in which Gmax and Gmed function was in an isometric, stabilising state. Contradictory findings from our study and Boren et al. (2011) might be attributed to the position of executing the side-plank hip abduction exercises (the lowermost leg flexed at the hip and knee versus the shoulders, hips and knees in line bilaterally). Furthermore, the differences in methodology, such as the study population (high-performance versus healthy individuals) and joint position angles used to execute this exercise (35° of hip abduction range versus abduction) could also have contributed to the difference.

Hip abduction is one of the essential requirements of field hockey. A player has to step to the side (a pure concentric hip abduction movement) to perform basic actions of field hockey, such as passing a ball, receiving a ball, shooting for goal and engaging in a tackle. Therefore, it was not unexpected that the %MVIC findings of Gmax and Gmed in high-performance female field hockey players were very high, considering the basic actions performed in field hockey.

### The single-leg squat

As presented in Table 2, our study found a 125.65%MVIC for Gmax and 126.30%MVIC for Gmed, whilst Boren et al. (2011) reported a 70.74%MVIC for Gmax and 82.26%MVIC for Gmed. As shown in Table 4, considering Gmax, there was no significant difference in activation between the side-plank hip abduction with the dominant leg at the bottom (*p* = 0.4807, *d* = −1.03) and side-plank hip abduction with the dominant leg on top (*p* = 0.3685, *d* = −1.31) when compared to the single-leg squat. However, there was a significant difference between the single-leg squat and plank with hip extension (*p* = 0.0487, *d* = 2.91). Concerning Gmed, there was no significant difference in activation amongst the side-plank hip abduction with the dominant leg at the bottom (*p* = 0.8837, *d* = −0.23), side-plank hip abduction with the dominant leg on top (*p* = 0.2606, *d* = −1.77) and plank with hip extension (*p* = 0.4240, *d* = 1.26) when compared to the single-leg squat. Boren et al. (2011) concluded that the single-leg squat was the fifth-best exercise to prescribe for strengthening Gmax and the third-best exercise to strengthen Gmed. On the contrary, we found that the single-leg squat elicited the highest %MVIC activation of both Gmax and Gmed compared to the other three exercises. This finding suggested that the greater movement complexity of the single-leg squat required that the body had to change the joint angles of the hip and knee joints during the execution of the action that resulted in greater %MVIC for both Gmax and Gmed.

In a systematic review by Macadam and Feser (2019), it was reported that differences in %MVIC of the single-leg squat across studies might be attributed to differences in the depth of the squat, the position of the contralateral leg and the experience of the participant with the execution of the exercise. Ayotte et al. (2007) studied a single-leg wall squat and highlighted that the free leg should be extended from the knee to elicit a higher %MVIC of Gmax. Similarly, in our study, the contralateral (free) leg was extended. Furthermore, we replicated the position described by Boren et al. (2011) that required the participants to execute the single-leg squat by touching a chair of 47 cm in height with their buttocks. Each participant’s dominant leg was 15 cm away from the chair. Boren et al. (2011) failed to specify the distance their participants had to stand from the chair.

When all four body weight rehabilitation exercises are considered, the single-leg squat required the most balance, which results in greater activation of Gmax. Macadam and Feser (2019) stated that a unilateral version of a vertically orientated exercise, such as a single-leg squat, results in greater Gmax %MVIC compared to the bilateral squat version. The imposed demands of this vertically orientated exercise result in Gmed functioning in its primary role as a hip abductor, whereas Gmax functions in its secondary role as a hip abductor to maintain stability of the pelvis. Furthermore, Gmax functions as a hip lateral rotator to minimise knee valgus collapse resulting from hip internal rotation and adduction (Distefano et al. 2009; Macadam et al. 2015; Reiman et al. 2012). Rainsford (2015) confirmed this by stating that the vertical force vector in which the single-leg squat is executed may place increased demands on the gluteal muscles to maintain lower extremity alignment. Lee and Jo (2016) stated that activation of muscles of the lower extremity differs according to the size of the base of support, as well as the height of the centre of gravity.

Moreover, movement of the pelvis away from the body’s base of support requires higher activation of all muscles around the hip, especially Gmed (Ayotte et al. 2007; Reiman et al. 2012). Against this background, it could be concluded that high Gmax and Gmed activity is generated because of the base of support being shifted away from the body’s centre of gravity. Furthermore, the gravitational forces result in substantial hip adduction torque during the single-leg squat that Gmed must resist to maintain lower extremity alignment (Distefano et al. 2009). It explains the greater activation of Gmed to stabilise the pelvis and knee in this position. The high level of activation is typical as it involves all the major functions of Gmax and Gmed, such as balancing on a single limb, stability of the lumbopelvic region and control over isotonic movements, eccentrically during hip flexion and concentrically during hip extension (Distefano et al. 2009).

Squatting applies in all actions of field hockey, especially whilst tackling and receiving the ball (Wege et al. 2006). Therefore, given that this action is often performed in field hockey, it is not surprising that the %MVIC levels of Gmax and Gmed in this high-performance population are higher than those of other studies. Biokineticists and conditioning coaches should use discretion when female field hockey players undertake this advanced exercise. Of the four body weight rehabilitation exercises examined, this is the exercise that can result in the most compensatory movements, such as an anterior pelvic tilt or hip adduction and femoral internal rotation, which result in knee valgus. Modifications and progression of the exercise should always be in accordance with the capabilities of the player. Proper balance and lower extremity alignment are the predominant requirements for the execution of the single-leg squat.

### Plank with hip extension

Given that hip extension is the primary function of Gmax, exercises that involve hip extension are typically prescribed for strengthening of this muscle (Macadam & Feser 2019; Neumann 2010; Rainsford 2015; Van Putte et al. 2014). Boren et al. (2011) was the only study that conducted this exercise in a plank version. As presented in Table 2, our study found a 122.73%MVIC for Gmax and 125.04%MVIC for Gmed, whilst Boren et al. (2011) reported a 106.22%MVIC for Gmax and 75.13%MVIC for Gmed. The high activation levels of Gmax found by both Boren et al. (2011) and our study imply that greater Gmax recruitment is required when an individual has less ground contact points and is only supported by one foot and both elbows (Macadam & Feser 2019). The 106.22%MVIC exhibited by Gmax in the study by Boren et al. (2011) was the highest reported for this muscle.

Additionally, Macadam and Feser (2019) concluded that plank with hip extension is the best body weight exercise for strengthening Gmax. In contrast, we found that the plank with hip extension elicited the lowest Gmax activation and is, therefore, the fourth-best exercise to prescribe for strengthening Gmax in high-performance female field hockey players. As shown in Table 4, concerning Gmax, we found no significant difference in activation between the side-plank hip abduction with the dominant leg at the bottom (*p* = 0.2000, *d* = 1.88) and side-plank hip abduction with dominant leg on top (*p* = 0.2770, *d* = 1.60) when compared to the plank with hip extension. However, a significant difference was found between the single-leg squat and plank with hip extension (*p* = 0.0487, *d* = 2.91). With regards to Gmed, plank with hip extension was found to be the third-best exercise for strengthening this muscle. Furthermore, there was no significant difference in activation amongst the side-plank hip abduction with the dominant leg at the bottom (*p* = 0.5134, *d* = 1.03), side-plank hip abduction with the dominant leg on top (*p* = 0.7437, *d* = −0.52) and the single-leg squat (*p* = 0.4240, *d* = 1.26), compared to the plank with hip extension.

Considering the basic actions performed in field hockey, it was not surprising that the %MVIC findings of Gmax and Gmed in this high-performance population were higher than those reported in other studies. As noted, squatting and side-stepping actions in field hockey require muscle activity of both Gmax and Gmed. Furthermore, performing lunge actions to engage in a tackle or to pass the ball, as well as the kick back action when running with the ball, are all examples of actions in field hockey that involve hip extension. Given that hip extension is the primary function of Gmax, the very high %MVIC generated from the plank with hip extension exercise in field hockey players can be attributed to the exceptional demands of field hockey in this position. Therefore, strengthening a player in this position is crucial for optimal performance.

It is important to note that compensation, such as lumbar hyperlordosis, can result if the player lacks adequate core stability (Jeong et al. 2015). Therefore, biokineticists and conditioning coaches should first ensure that the player has a high degree of core stability before introducing the plank with hip extension. If this exercise is included in the prehabilitation programme, the practitioner should remain vigilant for this type of compensation. Sufficient core stability is the absolute requirement for the plank with hip extension exercise.

### Summary of exercise effect on the Gmax and Gmed

A key observation in our study was that the four exercises investigated produced rather similar Gmax (*p* = 0.2558) or Gmed (*p* = 0.6285) activation, as shown in Table 3. Concerning the estimated effect sizes (ω = 0.10 for Gmax), which can be interpreted as the size of a correlation, the effect of ‘exercise’ on Gmax can be considered as low. It could, therefore, be concluded that there was only a small variation in Gmax activation between the four body weight rehabilitation exercises. Hence, the direction in which force was applied during the single-leg squat (closed kinetic chain) and plank with hip extension (open kinetic chain) significantly influenced the %MVIC of Gmax. We found that the closed kinetic chain single-leg squat is distinctly superior to the open kinetic chain plank with hip extension, concerning the activation of the Gmax in high-performance female field hockey players. However, this phenomenon was not noted in Gmax activation between the two other open kinetic chain exercises, namely side-plank hip abduction with the dominant leg at the bottom and side-plank hip abduction with dominant leg on top.

With regards to Gmed, the estimated effect size was zero (Table 3), suggesting that Gmed exhibited similar levels of activation during all four body weight rehabilitation exercises amongst the participants.

Our study was conducted exclusively on high-performance female field hockey players, therefore limiting generalisation to this group as it only provided data on the participants’ dominant side. The use of sEMG is associated with limitations because of various influences on the sEMG signal amplitude such as cross-talk (Bishop et al. 2018; Cochrane et al. 2019; Serner et al. 2014). Owing to the close proximity of the TFL muscle and gluteus minimus to Gmed, it is possible that these two muscles may contribute to the muscle activity recorded by the sEMG signal for Gmed (Distefano et al. 2009). We attempted to minimise this phenomenon by using standardised methods for electrode placement, securing the electrodes with adhesive tape to inhibit movement and by detecting the sEMG signal output before data collection.

It is theorised that when a specific primary muscle responsible for a particular joint movement weakens, the synergistic muscle becomes the new primary muscle responsible for the movement (Bishop et al. 2018; Boren et al. 2011; Lee & Jo 2016). The TFL is a muscle that is synergistic with Gmed and assists Gmed during abduction of the hip (Bishop et al. 2018; Han et al. 2018). Future studies should consider the influence of the TFL muscle as a synergistic muscle of Gmed and determine the gluteal-to-TFL muscle activation (GTA index) by comparing sEMG muscle activation of Gmax, Gmed and the TFL in executing the four body weight rehabilitation exercises of our study that have been designed to target Gmax and Gmed.

## Conclusion and practical implications

To optimise the performance of high-performance female field hockey players, biokineticists and conditioning coaches should select the body weight rehabilitation exercise that elicits the highest %MVIC that will result in Gmax and Gmed strength improvement. Given that our study is the first of its kind to examine the effect of these exercises on high-performance female field hockey players, biokineticists and conditioning coaches can benefit by incorporating the findings into the programme prescription during prehabilitation or the later stages of rehabilitation for this population. The conditioning coach, in particular, can benefit from the findings presented here, given that these exercises can be performed on the playing pitch as part of a warm-up without the need for any equipment.
